# Marital Quality and Heavy Alcohol Use Among Older Couples

**DOI:** 10.1093/geroni/igab046.1569

**Published:** 2021-12-17

**Authors:** Angela Curl, Jennifer Bulanda, Amy Restorick Roberts

**Affiliations:** Miami University, Oxford, Ohio, United States

## Abstract

Supportive marital relationships may reduce partners’ problematic health behaviors, whereas unhappy relationships may lack efficacious spousal monitoring of health and increase the likelihood of using maladaptive coping strategies, such as heavy alcohol use, to deal with relationship problems. We used pooled data from the 2014 and 2016 waves of the Health and Retirement Study to examine how both partners’ perceptions of marital quality were associated with heavy drinking. Our analytic sample included married couples in which both spouses were over age 50, completed the leave-behind psychosocial questionnaire, and provided non-missing data on marital quality and alcohol use (n=2,095 couples). Measures included both positive and negative dimensions of marital quality and controls for sociodemographic, economic, health, household and marital characteristics. Using Proc Glimmix, we estimated a dual-intercept Actor-Partner Interdependence Model (APIM), in which separate equations were computed simultaneously for husbands and wives. For husbands, higher negative marital quality was associated with an increase in the odds of their own heavy drinking (OR=1.27), but there was no significant association between wives’ marital quality and husbands’ heavy drinking behavior. For wives, marital quality was not significantly associated with their own heavy drinking, but husbands’ higher ratings of both negative and positive marital quality increased the risk of wives’ heavy drinking (OR=1.60 and OR=1.75, respectively). Results suggest that marital quality is associated with heavy drinking in later life: self-ratings of marital quality matter for men, whereas spousal perceptions of marital quality are more important for women.

